# Histone demethylase KDM3B protects against ferroptosis by upregulating SLC7A11

**DOI:** 10.1002/2211-5463.12823

**Published:** 2020-03-18

**Authors:** Yishu Wang, Yao Zhao, Haihua Wang, Chengliang Zhang, Meiqi Wang, Yong Yang, Xin Xu, Zhenbo Hu

**Affiliations:** ^1^ Laboratory for Stem Cell and Regenerative Medicine The Affiliated Hospital of Weifang Medical University Weifang China; ^2^ College of Bioscience and Technology Weifang Medical University Shandong China

**Keywords:** ATF4, ferroptosis, histone, histone demethylase, KDM3B, SLC7A11

## Abstract

Ferroptosis is a type of adaptive cell death driven by cellular metabolism and iron‐dependent lipid peroxidation. Though multiple genes (including SLC7A11 and GPX4) have been demonstrated to play key roles in ferroptosis, little is known about the epigenetic regulation of this process. Here, we report that KDM3B, a histone H3 lysine 9 demethylase, can protect against ferroptosis induced by Erastin, an inhibitor of SLC7A11. KDM3B overexpression in HT‐1080 cells results in decreased histone H3 lysine 9 methylation. Furthermore, KDM3B upregulates the expression of SLC7A11 through cooperation with the transcription factor ATF4. In summary, we identify here KDM3B as a potential epigenetic regulator of ferroptosis.

## Graphical abstract

KDM3B protects cells from Erastin‐induced ferroptosis through transcriptional upregulation of SLC7A11 via interaction with ATF4. Therefore, small molecule targeting of KDM3B can resensitize Erastin‐resistant cancer cells to ferroptosis induction and thus may have potential as a possible strategy to induce ferroptosis‐associated cancer cell death.

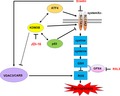

**Abbreviations**
AML: acute myeloid leukemia
GPX4: glutathione peroxidase 4
RSL3: Ras‐selective lethal small molecule 3

Ferroptosis is a newly discovered form of regulated cell death characterized morphologically by the abnormal mitochondria structure and mechanically by the iron‐dependent accumulation of lipid hydroperoxides to lethal concentrations 1. Ferroptosis dysfunctions have been demonstrated to be associated with multiple physiological and pathological processes, including cancer cell death, neurotoxicity, neurodegenerative diseases, acute renal failure, drug‐induced hepatotoxicity, hepatic and heart ischemia/reperfusion injury, and T‐cell immunity. A large number of genes play essential roles in ferroptosis. For example, glutathione peroxidase 4 (GPX4) functions as a negative regulator of ferroptosis by limiting ROS production. SLC7A11, a specific light‐chain subunit of the cystine/glutamate antiporter, also plays a negative role in ferroptosis. Thus, ferroptosis can be activated by SLC7A11 inhibition with the use of Erastin or by GPX inhibition with the use of Ras‐selective lethal small molecule 3 (RSL3) 2, 3.

Few details are known about the epigenetic regulation of ferroptosis. Lymphoid‐specific helicase (LSH), a DNA modifier, interacts with WDR76 to suppress ferroptosis by inducing lipid metabolism‐associated genes such as GLUT1‐ and ferroptosis‐related genes SCD1 and FADS2 4. A nuclear deubiquitinating enzyme, BAP1, represses SLC7A11 expression by decreasing H2A ubiquitination occupancy on the SLC7A11 promoter leading to elevated lipid peroxidation and in turn ferroptosis 5. H2B ubiquitination, on the other hand, is an epigenetic marker for SLC7A11 expression regulated by p53 6. Moreover, (+)‐JQ1, a BET family inhibitor, regulates ferritinophagy and the expression of ferroptosis‐associated genes by epigenetically inhibiting BRD4 through suppression of the histone methyltransferase G9a or activation of the histone deacetylase SIRT1 7.

Here, we reported the regulation of ferroptosis by histone demethylase KDM3B. KDM3B confers resistance to Erastin‐induced ferroptosis by activating the expression of SLC7A11, a key player of ferroptosis. Thus, we can surmise KDM3B as a new ferroptosis‐associated gene.

## Materials and methods

### Reagents and human cell lines

Erastin was purchased from Sigma (Shanghai, China), RSL3 was purchased from Selleckchem (Shanghai, China), and KDM3B inhibitor JDI‐16 was purchased from Topscience (Shanghai, China).

Human cell lines used in this study (HT‐1080, catalog number #ACC315, 293, catalog number #ACC305, and THP‐1, catalog number #ACC16) are held by the Leibniz‐Institute DSMZ‐German Collection of Microorganisms and Cell Cultures (Braunschweig, Germany). Cells were cultured using the recommended culture conditions (https://www.dsmz.de/). All cells were authenticated using the standard STR (short tandem repeats) genotyping method (ANSI/ATCC ASN‐0002‐2011) 8.

### Transient and stable transfection

The plasmids containing the CDS region of KDM3B (described previously 9) and JMJD1C (kindly provided by Kristian Helin 10) were inserted into pMCN‐GFP‐vector to construct pMCN‐GFP‐KDM3B/puromycin and pMCN‐GFP‐JMJD1C/puromycin, respectively. KDM3B siRNA was purchased from Qiagen (Shanghai, China).

Transient transfection of plasmids and siRNA was performed using Lipofectamine 2000 (Invitrogen, Shanghai, China) per the manufacturer's protocol. Stable transfection was performed as shown below. High viability cells were seeded at 2 × 10^5^ in 10 mL Dulbecco's modified Eagle's medium/10% FBS for single clone isolation on tissue culture dishes. After 16 h, the medium was reduced to 5 mL and transfected with the following superfect (Qiagen) protocol. 5 μg of each plasmid was used. The HT‐1080 and 293 cells were selected after a further 24 h with 0.4 μg·mL^−1^ puromycin (Invitrogen). After 14 days, the first puromycin‐resistant single clones should become visible.

### Cell proliferation assay

HT‐1080 and 293 cells were plated at the seeding density of 2000/mL in 100 μL medium in 96‐well plates. Chemicals were added after 24 h. Suspension cells THP‐1 were plated at the seeding density of 4000/mL in 100 μL medium in 96‐well round‐bottom plates, and chemicals were added after 24 h. Plates were incubated for 2–4 days at 37 °C in 5% CO_2_. Cells were then lysed with ViaLight Plus kit (Lonza, Cologne, Germany) according to the manufacturer's protocol, and a chemiluminescent signal was detected with a TECAN Spark 10M microplate reader (Crailsheim, Germany).

### Western blotting

Total cellular proteins isolated from parental HT‐1080 and KDM3B overexpressed HT‐1080 were run on 15% SDS/PAGE for electrophoresis. Antihistone H3 (#06‐755; Millipore, Darmstadt, Germany) and anti‐H3K9‐me1 (#07‐450; Millipore) antibodies were used for primary detection. As secondary antibodies, either anti‐rabbit or anti‐mouse IgG conjugated with horseradish peroxidase (HRP; GE Healthcare, Braunschweig, Germany) was used. Western Lightning Plus ECL (Perkin Elmer, Waltham, MA, USA) reagents were used for fluorescence production and AI600 (GE Healthcare, Shanghai, China) was used for fluorescence detection to visualize the proteins detected.

### Quantitative PCR

One million cells were collected for RNA isolation. Two micrograms of total RNA from HT‐1080 and 293 was reverse‐transcribed into cDNA using Invitrogen Superscript II reverse transcriptase (Life Technologies, Shanghai, China) according to the manufacturer's instructions. Random primers were used to obtain cDNA. Synthesized cDNA served as the template in 20 µL qPCRs. qPCR was performed using SYBR protocols (Takara, Dalian, China). The PCR was run in an ABI7500 fast real‐time PCR machine with qPCR cycling conditions of 95 °C for 30 s, followed by 40 cycles of 95 °C for 3 s and 60 °C for 30 s. Relative concentrations of each target template were calculated according to the comparative Ct method. Expression of target transcripts was standardized to GAPDH. qPCR analyses were performed in triplicate. Primers used are available upon request.

### Luciferase assay

SLC7A11 promoter was cloned from blood cDNA of healthy donors as described previously 11 and was inserted into pGL3‐enhancer plasmids (Promega, Madison, WI, USA). pRL‐TK (Promega) was used as control plasmid. Luciferase and renilla activities were detected using Promega Dual‐Luciferase Reporter Assay System. The assay was finished in Sirius L Tube luminometer (Berthold Detection Systems, Pforzheim, Germany).

### Statistical analysis

Quantitative results are reported as mean ± standard deviation (SD). The statistical analysis of cell proliferation, qPCR, and luciferase assays was performed using Student's *t*‐test. Comparisons with a *P* value ≤ 0.05 were considered statistically significant.

## Results and Discussion

### Overexpressed KDM3B prevents cells from Erastin‐induced ferroptosis

**Fig. 1:**
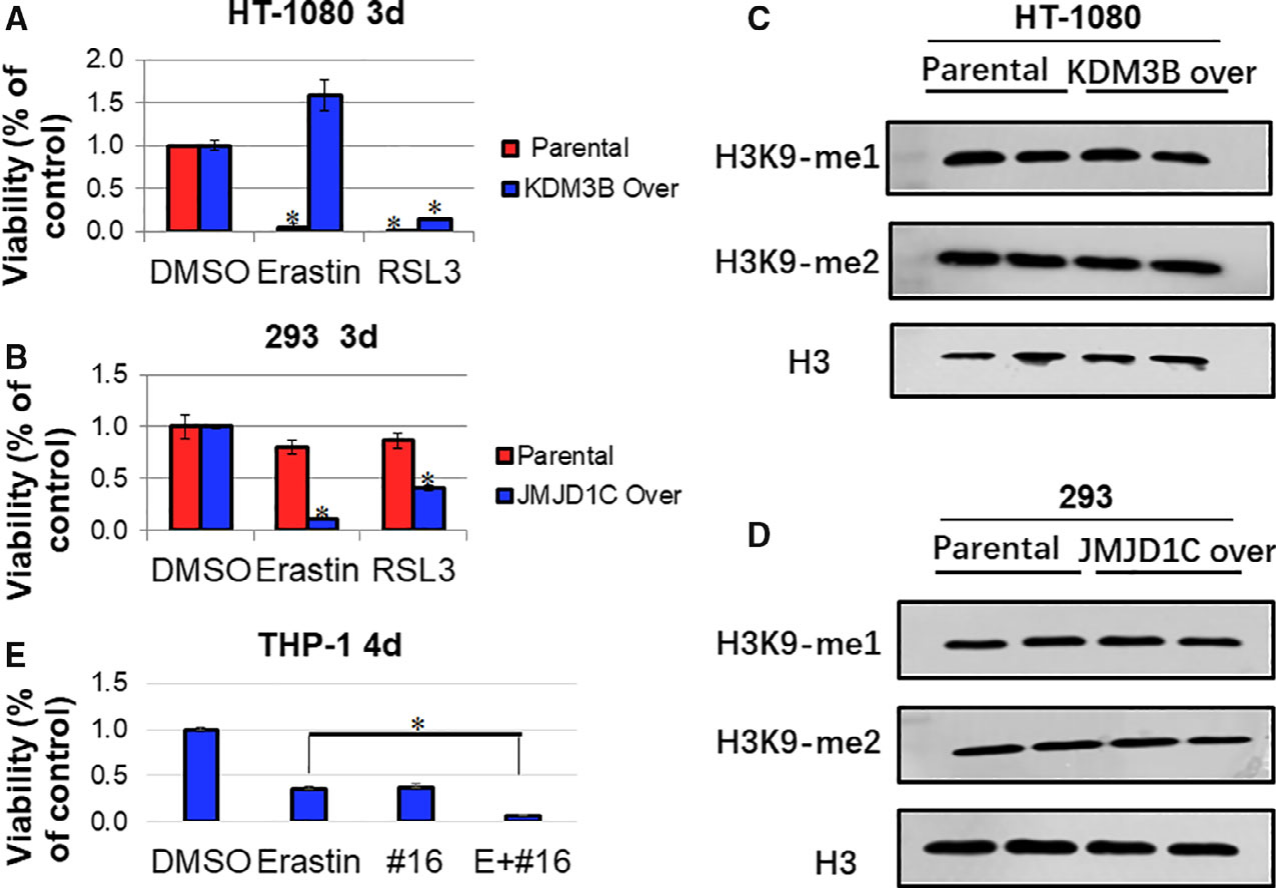
The effect of KDM3B overexpression on the Erastin‐induced ferroptosis. (A) The effect of KDM3B in HT‐1080 cells on the ferroptosis induced by Erastin (5 μm) and RSL3 (2.5 μm). KDM3B stably overexpressed HT‐1080 was constructed as described in the materials and methods. Parental and KDM3B over cells were plated in 96‐well plates at a concentration of 1500 cells per well and incubated with indicated compounds 24 h after plating. Three days later, cells were collected for proliferation detection. (B) The effect of JMJD1C in 293 cells on the ferroptosis induced by Erastin (5 μm) and RSL3 (2.5 μm). JMJD1C stably overexpressed 293 was constructed as described in the materials and methods. Parental and JMJD1C over cells were plated in 96‐well plates at a concentration of 1500 cells per well and incubated with indicated compounds 24 h after plating. Three days later, cells were collected for proliferation detection. (C) The effect of KDM3B stable overexpression on the H3K9 monomethylation and dimethylation of HT‐1080 cells. Parental and KDM3B over HT‐1080 cells were collected and lysed for Western blot detection. H3 was used as endogenous control. (D) The effect of JMJD1C stable overexpression on the H3K9 monomethylation and dimethylation of 293 cells. Parental and JMJD1C over HT‐1080 cells were collected and lysed for Western blot detection. H3 was used as endogenous control. (E) The effect of KDM3B inhibitor JDI‐16 on Erastin‐induced repression of cell proliferation of THP‐1 cells. THP‐1 cells were plated in 96‐well round‐bottom plates at a concentration of 4000 cells per well and incubated with indicated compounds (Erastin, 5 μm, JDI‐16, 12.5 μm) 24 h after plating. Four days later, cells were collected for proliferation detection. For (A, B, E), three independent experiments were performed, quantitative results are reported as mean ± SD, the statistical analysis was performed using Student's *t*‐test, and *P* value smaller than 0.05 (represented by *) was regarded as statistically significant.

Though very less is known about the epigenetic regulation of ferroptosis, we have been working on histone H3 lysine 9 demethylases KDM3B in hope of elucidating its mysteries. Our RNA sequencing results showed that potential inhibitors of KDM3B regulate ferroptosis‐associated genes, especially SLC7A11 12. We postulated that KDM3B and its family member JMJD1C may be involved in ferroptosis. HT‐1080 is a classical cell line for ferroptosis research, and we stably expressed ectopic KDM3B in HT‐1080 cells. We then treated HT‐1080 cells overexpressing KDM3B with type I ferroptosis inducer Erastin and type II ferroptosis inducer RSL3. As shown in Fig. 1A, KDM3B overexpression in HT‐1080 yields robust resistance to Erastin‐ but only modest resistance to RSL3‐induced cell death. We also examined the effect of JMJD1C on Erastin‐induced ferroptosis by using 293 cell line with stable JMJD1C overexpression. As shown in Fig. 1B, JMJD1C, a KDM3B family member, does not confer Erastin resistance. Since KDM3B is a histone demethylase, we also measured the effect of stably overexpressed KDM3B on the methylation modifications of histone H3 lysine 9. In contrast with transient enforced expression of KDM3B, stably overexpressed KDM3B showed no significant effect on H3K9 methylation levels (Fig. 1C). JMJD1C did not significantly change H3K9 methylation levels (Fig. 1D). The balanced histone methylation levels after stable transfection served as the prevailing explanation for the lack of differential H3K9 methylation detection. Another plausible reason is that KDM3B may catalyze other histone modifications reported previously 13 or nonhistone substrates 14. To sum up, we postulated that KDM3 family member KDM3B negatively regulates ferroptosis.

To further study the role of KDM3B in ferroptosis, we also measured the effect of KDM3B inhibitors on Erastin‐induced cell proliferation repression in cells different from HT‐1080. Yang *et al*. 2 examined the effect of Erastin on 117 cell lines and identified Erastin‐sensitive and Erastin‐insensitive cell lines. Notably, acute myeloid leukemia (AML) cells are insensitive to Erastin (AUC > 5.5, the area under the concentration–response curve). Since KDM3B has been shown to associate with AML 15, 16, we treated Erastin‐resistant AML cell line THP‐1 (as reported by Yang *et al*.) with Erastin and KDM3B inhibitor JDI‐16. As shown in Fig. 1E, JDI‐16 synergized with Erastin to repress the cell proliferation of THP‐1. These results showed that KDM3B inhibition could sensitize Erastin‐resistant cancer cells for ferroptosis‐associated cell death.

### KDM3B activates the expression of SLC7A11

**Fig. 2:**
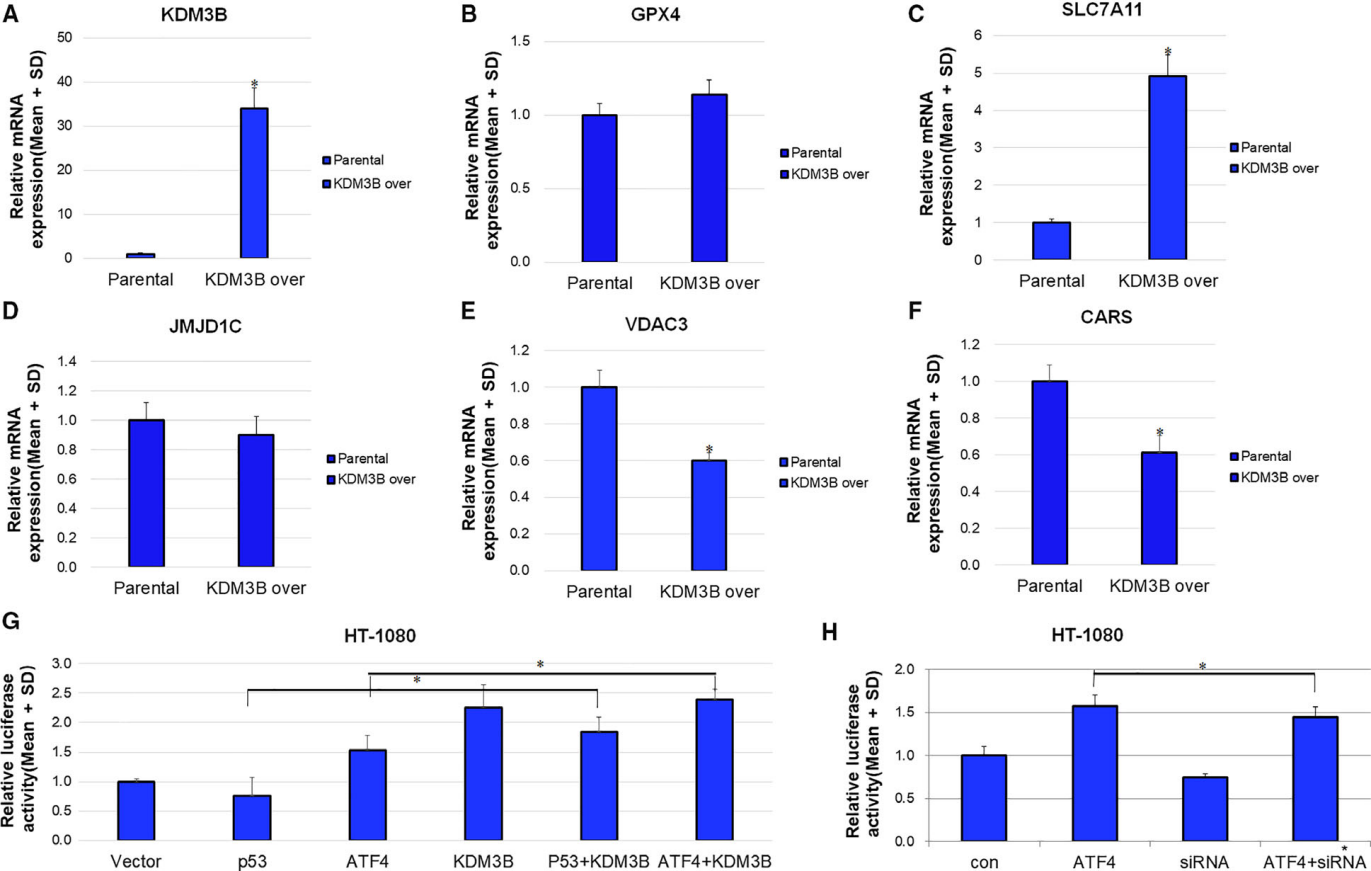
The transcriptional regulation of SLC7A11 by KDM3B. (A–F) The mRNA levels of indicated genes affected by KDM3B overexpression. Parental and KDM3B overexpressed HT‐1080 cells were collected for RNA extraction and qPCR. GAPDH was used as an endogenous control. (G) The effect of enforced ATF4, p53, KDM3B, and the combination on the SLC7A11 promoter activities. The SLC7A11 promoter reporter along with ATF4, p53, and KDM3B plasmids was transfected into HT‐1080 cells with pRL‐TK as an endogenous control. (H) The effect of enforced ATF4 and KDM3B siRNA on the SLC7A11 promoter activities. The SLC7A11 promoter reporter along with ATF4 and KDM3B siRNA was transfected into HT‐1080 cells with pRL‐TK as an endogenous control. For (A–H), three independent experiments were performed, quantitative results are reported as mean ± SD, the statistical analysis was performed using Student's *t*‐test, and *P* value smaller than 0.05 (represented by *) was regarded as statistically significant.

**Fig. 3:**
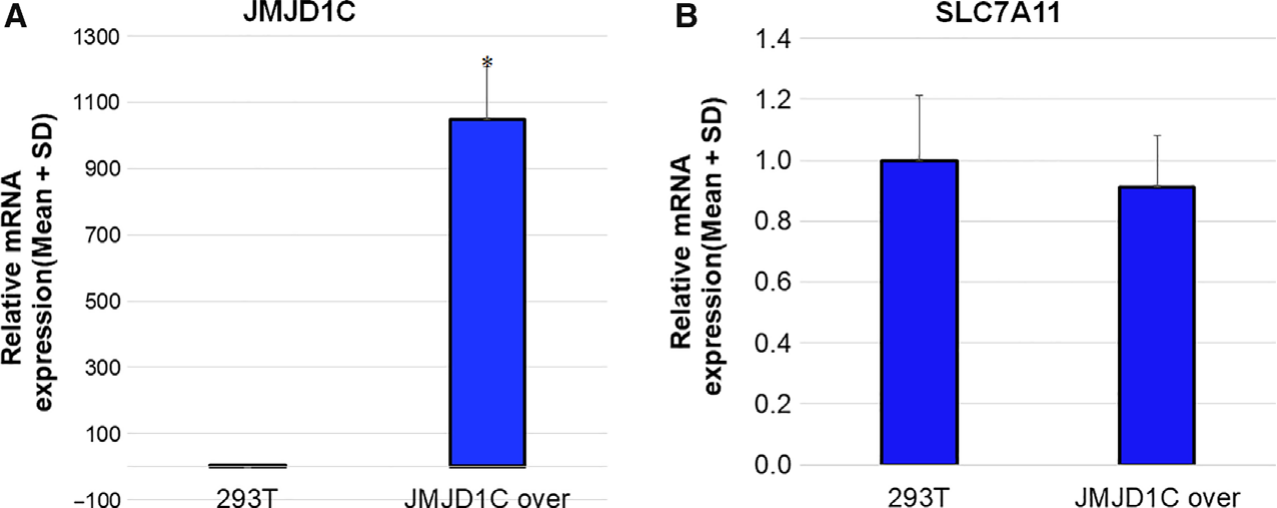
The effect of JMJD1C overexpression on SLC7A11 mRNA. (A) JMJD1C and (B) SLC7A11 mRNA levels after JMJD1C transfection into 293T cells. 293T cells were transfected with JMJD1C plasmids as described in materials and methods and collected for RNA extraction and qPCR. GAPDH was used as an endogenous control. Three independent experiments were performed. Quantitative results are reported as mean ± SD. The statistical analysis was performed using Student's *t*‐test. *P* value smaller than 0.05 (represented by *) was regarded as statistically significant.

Since KDM3B confers robust resistance to Erastin and modest resistance to RSL3, we measured the mRNA changes of key ferroptosis‐associated genes upon KDM3B overexpression. As shown in Fig. 2A–F, multiple ferroptosis‐associated genes were affected by KDM3B overexpression in HT‐1080 cells. Among them, SLC7A11 showed significant upregulation by enforced KDM3B, consistent with the results that KDM3B bestow much more resistance to Erastin than RSL3. Moreover, JMJD1C overexpression had no discernible effect on SLC7A11 expression (Fig. 3), consistent with the aforementioned results that JMJD1C overexpression does not lead to Erastin resistance. Considering that SLC7A11 mediates the effect of multiple chromatin‐associated proteins including BAP1 and BRD4 on Erastin‐induced ferroptosis 5, 7, we thus postulated that KDM3B plays a role in the transcriptional regulation of SLC7A11.

Multiple transcriptional factors like ATF4 and p53 have been reported to positively and negatively regulate the expression of SLC7A11, respectively 3, 17. We measured the effect of KDM3B on the ATF4‐ or p53‐dependent luciferase activities of SLC7A11 promoter. As shown in Fig. 2G, KDM3B cooperates with ATF4 to activate the SLC7A11 promoter, whereas KDM3B rescues SLC7A11 repression by p53. On the other hand, ATF4‐activated elevation of SLC7A11 promoter activities could be attenuated by KDM3B knockdown (Fig. 2H). KDM3B may function as a coregulator of ATF4 or p53 for SLC7A11 regulation. ATF4 has been shown to interact with another KDM family member KDM4C 18. Additionally, ATF4 could be regulated by methylation catalyzed by PRMT1 19. P53 methylation has also been broadly reported for the determination of p53 function 20. KDM3B may regulate SLC7A11 in a manner independent of histone demethylation, but dependent on the demethylation of nonhistone substrates like ATF4 and or p53.

KDM3B could also regulate Erastin‐induced ferroptosis dependent on other genes such as VDAC3 and CARS 1. Erastin has been reported to induce ferroptosis through directly binding to VDAC2/3 leading to the alternation of the permeability of the outer mitochondrial membrane and the decreases of the rate of NADH oxidation 21. Loss of CARS, the cysteinyl‐tRNA synthetase, blocks Erastin‐induced ferroptosis 22. Both VDAC3 and CARS function as positive regulators of ferroptosis 23 and KDM3B could confer ferroptosis resistance by downregulating VDAC3 and CARS as shown in Fig. 2.

**Fig. 4:**
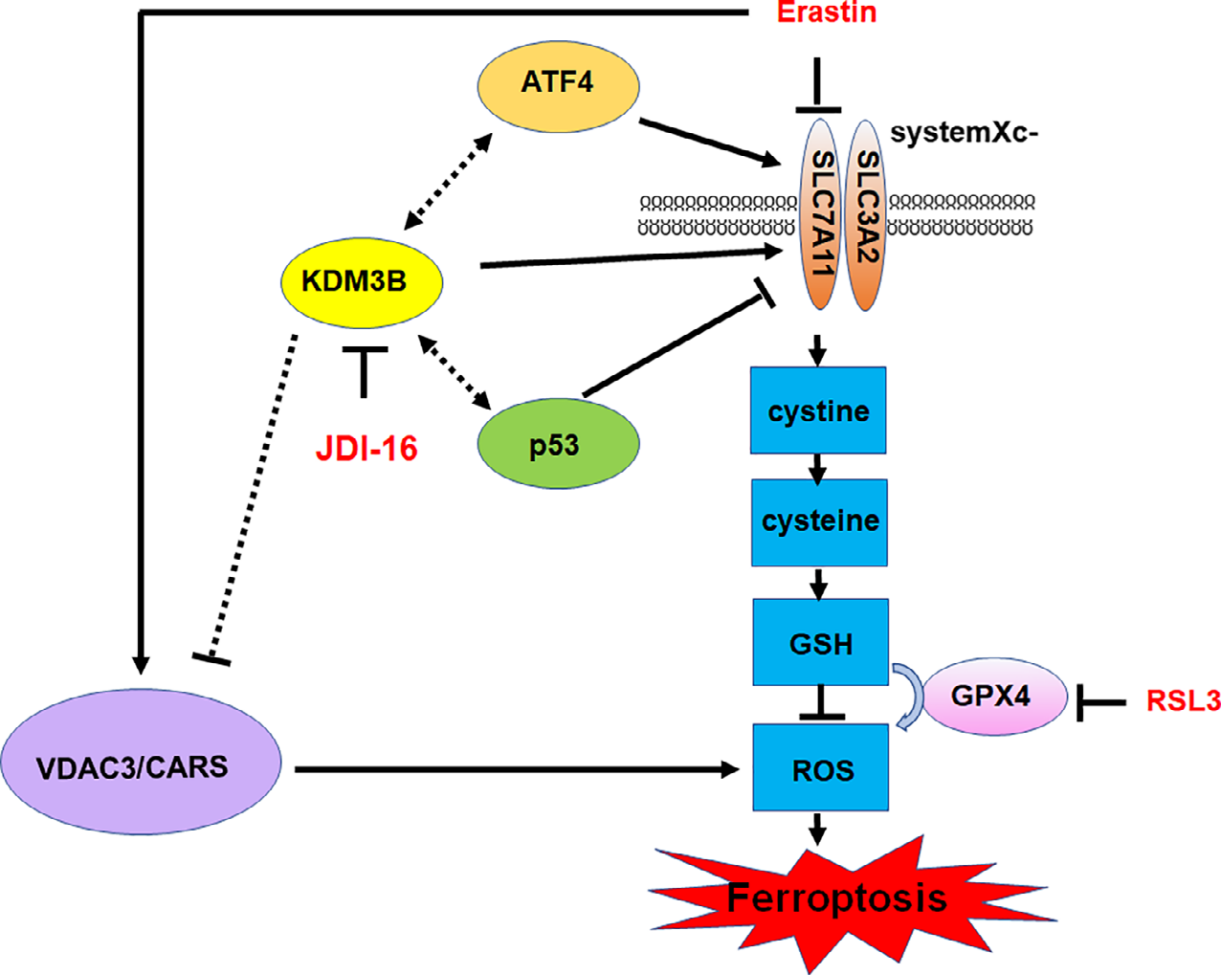
Schematic representation of the proposed model that characterizes KDM3B's role in Erastin‐induced ferroptosis. Class I FIN (ferroptosis inducer) Erastin and class II FIN RSL3 induce ferroptosis dependent on SLC7A11 and GPX4, respectively. KDM3B transcriptionally upregulates SLC7A11, probably through ATF4 and p53, to confer ferroptosis resistance. KDM3B also represses VDAC3 and CARS. KDM3B inhibitor JDI‐16 could resensitize cells to Erastin‐induced proliferation repression.

In conclusion, we reported the regulation of ferroptosis by KDM3B (Fig. 4). Further study of KDM3B regulation of ferroptosis in leukemia is merited.

## Conflicts of interest

All authors declare no conflict of interest.

## Author contributions

WY performed most experiments and drafted figures. ZY performed cell culture; WH performed cell proliferation detection. ZC and WM performed Western blotting. YY performed qPCR. XX supervised the project and drafted the manuscript. HZ supervised the project and modified the manuscript.
